# Anthrax protective antigen is a calcium-dependent serine protease

**DOI:** 10.1080/21505594.2018.1486139

**Published:** 2018-07-27

**Authors:** Lisanne Storm, Floris J. Bikker, Kamran Nazmi, Albert G. Hulst, Debora V. der Riet-Van Oeveren, Enno C.I. Veerman, John P. Hays, Wendy E. Kaman

**Affiliations:** a Department of Medical Microbiology and Infectious Diseases, Erasmus University Medical Centre, Rotterdam, The Netherlands; b Department of Oral Biochemistry, Academic Centre for Dentistry Amsterdam, University of Amsterdam and VU University Amsterdam, Amsterdam, The Netherlands; c Department of CBRN Protection, Netherlands Organization for Applied Scientific Research TNO, Rijswijk, The Netherlands

**Keywords:** Protective antigen, *B. anthracis*, calcium, serine protease, ANTXR1/2 receptor

## Abstract

*Bacillus anthracis* secretes a three component exotoxin-complex, which contributes to anthrax pathogenesis. Formation of this complex starts with the binding of protective antigen (PA) to its cellular receptor. In this study, we report that PA is a calcium-dependent serine protease and that the protein potentially uses this proteolytic activity for receptor binding. Additionally our findings shed new light on previous research describing the inhibition of anthrax toxins and exotoxin formation. Importantly, inhibition of the proteolytic activity of protective antigen could be a novel therapeutic strategy in fighting *B. anthracis-*related infections.

## Introduction

*Bacillus anthracis*, the causative agent of anthrax, secretes a three component exotoxin, *i.e*. lethal toxin, which consists of the cell binding protective antigen (PA), and the enzymes edema factor (EF) and lethal factor (LF). This lethal toxin penetrates host cells and is known to contribute to anthrax pathogenesis, making it a commonly used target in the development of vaccines and anti-infectives against this bacterial pathogen [1,2]. In order to penetrate the host cell, anthrax lethal toxin utilizes a multi-step mechanism. First, PA (PA_83_, 83 kDa) interacts with the host cell surface via at least two receptors, tumor endothelial marker 8 (TEM8/ANTXR1) and capillary morphogenesis gene 2 (CMG2/ANTXR2) [3,4]. Upon receptor binding PA_83_ is cleaved into PA_63_ (63 kDa) by furin, a calcium-dependent serine protease, which is produced by the host. PA_63_ subsequently forms a heptameric structure, known as a ‘pre-pore’, which interacts with the toxins LF and EF. The entire receptor-toxin complex is then endocytosed and transported into low-pH endosomes. Triggered by the acidic environment a pore forms across the endosomal membrane, after which EF and LF are released into the cytosol of the host cell [5]. Once present in the cell cytosol, EF and LF damage the cell in various ways; LF is a zinc-dependent metalloproteinase that cleaves and inactivates the mitogen-activated protein kinase (MAPK) kinases 1–4, 6 and 7 [6]. The cleavage events result in inactivation of the MAPK pathways thereby hampering a variety of cellular responses including cell division, apoptosis, and survival [7]. EF is a calmodulin-dependent adenylyl cyclase that elevates intracellular cAMP levels by converting ATP to cAMP, the classical second messenger, thereby causing diverse adverse side-effects [8]. These processes allow *B. anthracis* to evade the hosts` immune system and facilitate proliferation, resulting in the characteristic disease symptoms of anthrax that frequently lead to death. In this respect, understanding the first step in toxin formation, *i.e*. the interaction between PA and its receptor, is critical for the development of effective treatment strategies against *B. anthracis* related infections. In previous research, it has been shown that the interaction between PA_83_ and its receptors resembles an pH-dependent α-integrin/ligand binding interaction [9,10]. Additionally, Bradley and co-workers showed that EDTA inhibits the binding of PA_83_ to its receptor, suggesting a role of divalent cations in receptor binding [3]. The binding-associated regions of both PA_83_ and its receptors were identified [11], though the exact mechanism by which PA_83_ binds to its receptors remains to be elucidated.

In this study, we demonstrated that PA_83_ exhibits a hitherto unknown proteolytic activity which potentially plays a crucial role in the binding of PA_83_ to the ANTXR1 receptor.

## Results

**Figure 1.**
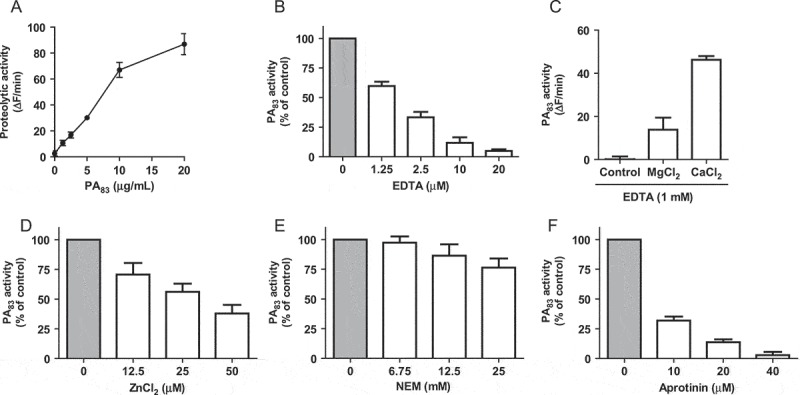
Biochemical characterization of PA_83_ proteolytic activity. PA_83_ proteolytic activity was determined using FRET-peptide PEK-054 with varying PA_83_ concentrations (A). The effect of varying concentrations of (specific) protease inhibitors and metal ions on the hydrolysis of 16 µM FRET-peptide PEK-054 by PA_83_ (20 μg/mL). PA_83_ activity without the addition of inhibitor or metal ions was taken as the ‘normalized’ (100%) value (B-G). Results are expressed as mean ± SEM (*n* = 3).

**Figure 2.**
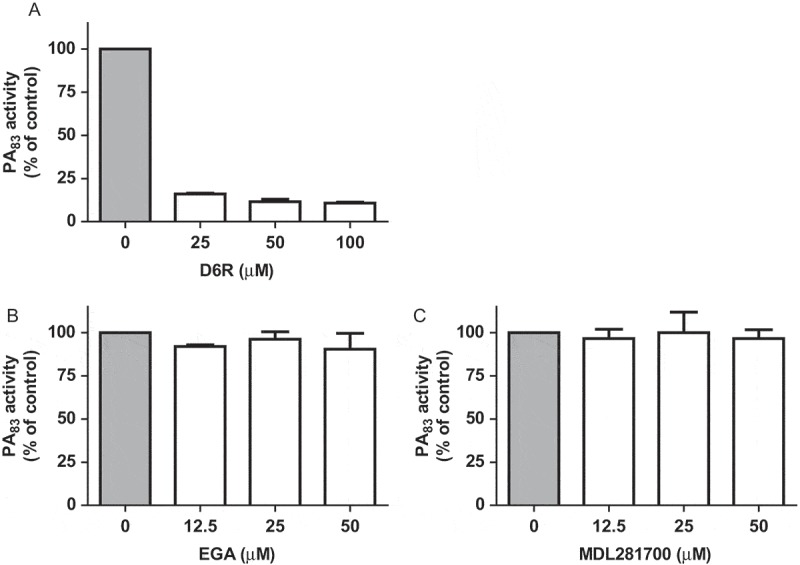
Effect of inhibitors of furin protease activity and lethal toxin formation on PA_83_ proteolytic activity. Varying concentrations of furin inhibitor D6R (A) and lethal toxin formation inhibitors EGA (B) and MDL28170 (C) were incubated with FRET-peptide PEK-054 and PA_83_ (20 μg/mL). PA_83_ activity without the addition of inhibitor was taken as the ‘normalized’ (100%) value. Results are expressed as mean ± SEM (*n* = 3).

PA_83_ proteolytic activity was characterized by measuring the dose-dependent degradation of the PEK-054 substrate using HEPES-buffered saline as reaction buffer (Figure 1(A)). No proteolytic activity could be detected when the experiment was performed in phosphate buffered saline (PBS) buffer (data not shown). In view of the calcium-chelating properties of phosphate ions, it was hypothesized that calcium ions are required for the proteolytic activity of PA_83_. This was corroborated by the finding that EDTA inhibited the proteolytic activity in a dose-dependent manner (Figure 1(B)). PA_83_ activity could be restored by the addition of Ca^2+^ and, to a lesser extent, Mg^2+^ ions (Figure 1(C)). In contrast, the addition of Zn^2+^ ions inhibited the proteolytic activity of PA_83_ (Figure 1(D)). Furthermore, we examined the effect of several (specific) protease inhibitors on PA_83_ activity. The addition of the cysteine protease inhibitor NEM hardly affected protease activity of PA_83_ (Figure 1(E)). In contrast, the serine protease inhibitor aprotinin at 40 µM virtually completely inhibited PA_83_ proteolytic activity (Figure 1(F)). Additional experiments showed that the furin inhibitor D6R was able to inhibit PA_83_ activity (Figure 2(A)). EGA and MDL281700, inhibitors of lethal toxin formation, showed no significant effect on the proteolytic activity of PA_83_ (Figure 2(B,C)).

**Figure 3.**
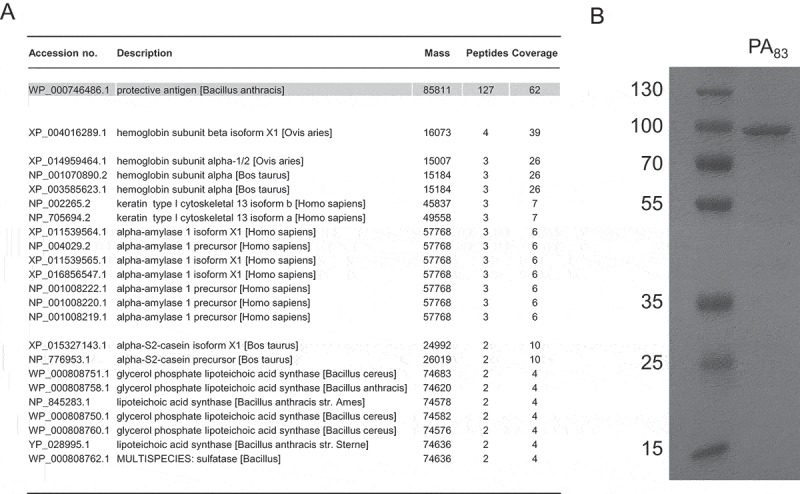
Purity analysis of the PA_83_ protein used within this study. LC MS/MS analysis of the PA_83_ protein. Search results of the Mascot Generic (MGF) files generated against the NCBI database using the Mascot search algorithm (Mascot 2.2.04) are shown. Only the proteins with ≥ 2 peptide hits are listed (A). SDS-PAGE analysis of the PA_83_ protein (2 µg) (B).

**Figure 4.**
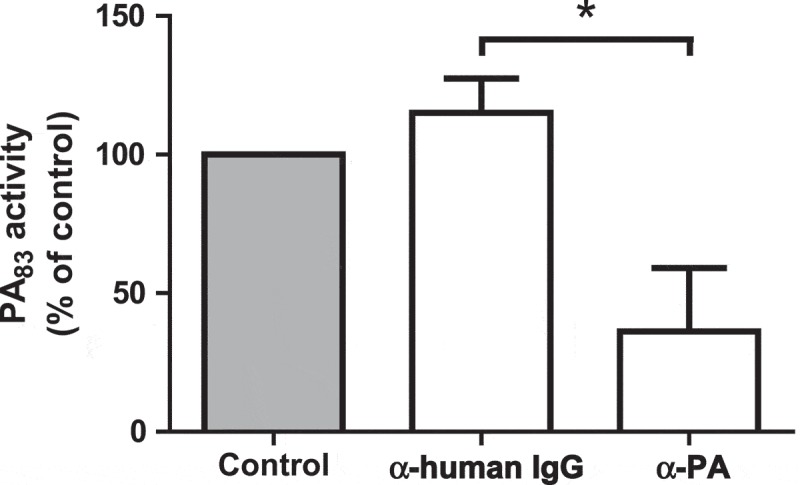
Specificity testing of the PA_83_ proteolytic activity. 20 µg/mL PA_83_ was pre-incubated with 15 µg/mL antibody α-PA/α-human IgG or PBS (control) for 5 hr. PA_83_ was measured using 16 μM FRET-peptide PEK-054. PA_83_ activity without the addition of antibodies was taken as the ‘normalized’ (100%) value. Results are expressed as mean ± SEM (*n* = 3). Significance was calculated using an unpaired, two-tailed students *t*-test (* *P*-value < 0.05).

Identity and purity studies of the purchased PA_83_ protein using LC MS/MS analysis and SDS-PAGE confirmed the identity of PA_83_. The intact, 83 kDa, protective antigen (coverage 62%) was not contaminated with any other proteolytically active proteins (Figure 3(A and B)). Additionally, inhibition of the proteolytic activity by a specific antibody for anthrax protective antigen (α-PA) confirms that the activity originates from the PA_83_ protein. No inhibition was observed upon addition of the antibody control (α-human IgG) (Figure 4).

**Figure 5.**
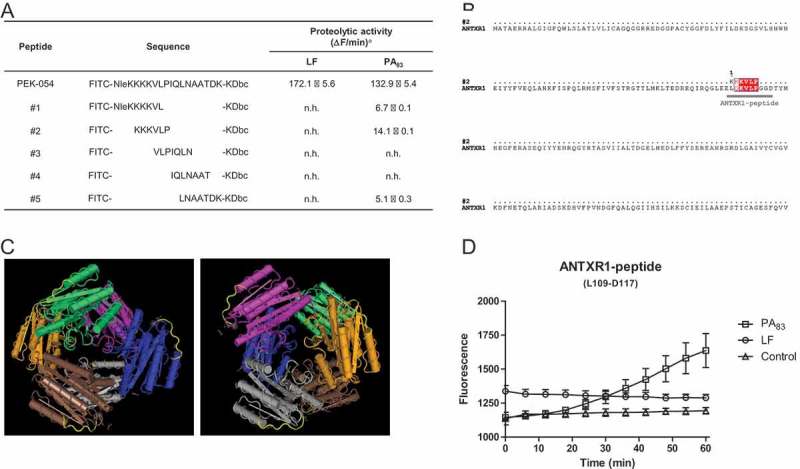
PA_83_ cleavage specificity and its potential role in ANTXR1 receptor binding. Hydrolysis of FRET-peptide PEK-054 and its derivatives using 20 µg/mL PA_83_ or LF *^a^* No hydrolysis: n.h. (A). ANTXR1 sequence alignment was performed using software programs ClustalW and ESPript 3.0 (Risler matrix, global score of 0.7). Sequence overlap between ANTXR1 and the peptide is depicted in red (B). Presence of the KVLP-region on the outside of the ANTXR1 protein is marked yellow. The 3D structure of the protein was generated using the Cn3D 4.3 program (C). Hydrolysis of ANTXR1 peptide (L109-D117) using 20 µg/mL PA_83_, 20 µg/mL LF or HEPES buffer (D). Results are expressed as mean ± SEM (*n* = 3).

To map the cleavage site for PA_83_, five FRET-based peptides, covering the full-length of PEK-054 (Figure 5(A)), were tested in the FRET assay with PA_83_ and LF, the protease for which the PEK-054 substrate was originally designed. Full-length PEK-054 was hydrolyzed by both proteins with comparable efficiency (LF: 172.1 ± 5.6 ΔF/min and PA_83_: 132.9 ± 5.4 ΔF/min). None of the PEK-054 derivatives was hydrolyzed by LF, however PA_83_ was able to hydrolyze derivatives #1, #2 and #5. Peptide #2 (FITC-KKKVLP-KDbc) was preferentially hydrolyzed by PA_83_ (14.1 ± 0.1 ΔF/min), followed by peptide #1 and #5 (6.7 ± 0.1 ΔF/min and 5.1 ± 0.3 ΔF/min respectively). No hydrolysis was observed with peptide #3 and #4 (Figure 5(A)). A BLAST search, followed by alignment analysis, revealed that peptide #2 has a homology region with the ANTXR1 receptor (Figure 5(B)). This region is present on the outside of each hexamer of the ANTXR1 receptor protein and is therefore accessible to proteases (Figure 5(C)). Based on the 4-amino acid sequence overlap between the ANTXR1 receptor and peptide #2 we designed a 9-amino acid containing FRET-peptide substrate based on the protein sequence of the ANTXR1 receptor protein (FITC-LQKVLPGGD-KDbc (L109-D117)/ANTXR1-peptide). Incubating this peptide with 20 μg/mL PA_83_ showed hydrolysis of the peptide with an average efficiency of 8.1 ± 1.8 ΔF/min (Figure 5(D)).

## Discussion

During a study on LF protease inhibitors we discovered that PA_83_, a protein known to be involved in pore formation during *B. anthracis* infection, possessed proteolytic activity on the FRET-peptide substrate to detect LF activity. This proteolytic activity is a feature of PA_83_ which has not been described before. Characterization experiments revealed that PA_83_ proteolytic activity depends on the presence of divalent cations. Besides, based on its response to specific protease inhibitors, it seems most plausible that PA_83_ belongs to the class of serine proteases. The strong cation dependency of PA_83_ explains why its proteolytic activity was not detected earlier. Besides, the fact that two Ca^2+^ ions are present in the quaternary structure of PA_83,_ strengthens the potential role of Ca^2+^ in PA_83_ protease activity [12]. In this respect, Bradley et al showed that binding of PA_83_ to its receptor is cation dependent whereas the binding interaction between the ANTXR1 receptor and PA_83_ is impaired in the presence of EDTA [3]. Additionally, Ramey et al showed that binding of PA_83_ to the ANTXR1 receptor is dependent on divalent cations, such as Mg^2+^ and Ca^2+^ [13]. Based on the above mentioned studies and our observation that not only calcium, but also magnesium, was able to restore PA_83_ proteolytic activity after inhibition with EDTA, we provide preliminary evidence that PA_83_ uses its proteolytic activity in order to facilitate its binding to the ANTXR1 receptor. *In silico* analysis revealed a sequence overlap between the substrate degraded with the highest efficiency, peptide #2 (FITC-KKKVLP-KDbc), and a region in the von Willebrand factor type A domain (vWA domain) of the ANTXR1 protein. A FRET-peptide substrate based on the sequence of this region (ANTXR1-peptide) was successfully degraded by PA_83,_ again suggesting that PA_83_ proteolytic activity plays a role in receptor binding. Further, results published by Fu et al showed that the region of the ANTXR1 receptor that overlaps with the sequence of peptide #2, interacts with the hydrophobic cleft of protective antigen [11].

The discovery of PA_83_ proteolytic activity sheds new light on previous research involving inhibitors of furin activity. Within lethal toxin formation this human protease cleaves PA_83_ into PA_63_ and PA_20_ after which PA_63_ forms a heptameric pre-pore. Like PA_83_, furin is a calcium dependent serine protease whose activity is inhibited in the presence of Zn^2+^ ions [14]. Further, the potential benefits of the use of furin inhibitors in the treatment of *B. anthracis* infection has already been proven *in vivo* [15–17]. However, the observed therapeutic effect of furin inhibitors may not only originate from furin inhibition but can also be related to inhibition of PA_83_ proteolytic activity. Here we show that there is indeed cross-inhibition of furin inhibitor D6R with PA_83_ proteolytic activity. Additionally, inhibitors of anthrax lethal toxin formation might work via another mechanism than suggested. For this reason we examined the effect of two compounds, EGA and calpain-inhibitor MDL281700, utilized in studies investigating the inhibition of lethal toxin formation, on the proteolytic activity of PA_83_ [18,19]. These compounds however did not affect PA_83_ proteolytic activity.

The results described in this study indicate that PA_83_ possesses proteolytic activity that potentially contributes to the binding of PA_83_ to its host cellular receptor. Additionally, PA_83_ is able to hydrolyze a protease substrate of which the sequence is based on MAPKK [20], suggesting that PA_83_ might possess LF-like activity. Thus it is hypothesized that by inhibiting the proteolytic activity of PA_83_ it may be possible to decrease the pathogenic potential of *B. anthracis*. In this light, the substrates described in this study could potentially help in future studies aiming to develop PA_83_ protease inhibitors. For example, the sequences of the peptide substrates utilized in this study could possibly be used to develop competitive inhibitors of PA_83_ protease activity, thereby reducing the efficiency of PA_83_ receptor binding or MAPKK degradation. However additional research is needed to precisely unravel the function of the PA_83_ proteolytic activity.

In conclusion, to the best of our knowledge, we are the first who describe proteolytic activity of PA_83_. This finding may lead to the development of novel therapeutic strategies against *B. anthracis* infection and virulence.

## Materials and methods

### Peptide synthesis

The fluorescence resonance energy transfer (FRET-) peptides used in this study were synthesized by solid-phase peptide synthesis using Fmoc chemistry and a MilliGen 9050 peptide synthesizer (Milligen-Biosearch). The peptides were flanked at their C-termini with a fluorescent probe (FITC) and flanked at their N-termini with a lysine coupled quencher (Dabcyl (Dbc)). FRET-peptides were purified by reverse-phase high-performance liquid chromatography (RP-HPLC). Purity of the FRET-peptides was confirmed using mass spectrometry, as described previously [21]. Stock solutions (800 µM) of the quenched FRET-peptides were prepared in DMSO and stored at – 20°C.

### Protease assay

Proteolytic activity was determined, as described earlier [22], by incubating 16 µM FRET-peptide with varying concentrations of PA_83_ or LF (both List Laboratories) in HEPES buffer (20 mM HEPES, 0.05% Tween-20, pH 8.2) using blackwell clear bottom 96-well plates (Corning). Plates were incubated at 37°C for 60 min, with fluorescence levels being recorded at 2 min intervals using a fluorescence microplate reader (FLUOstar Galaxy, BMG Laboratories) with an excitation wavelength of 485 nm and an emission wavelength of 530 nm. Fluorescence values were obtained after correction against the buffer control. Protease activity was defined in ΔF per minute (ΔF/min) [23]. An ΔF/min < 5 was considered as baseline, *e.g*. no hydrolysis (n.h.).

### Cleavage characteristics of PA_83_

20 µg/mL PA_83_ was incubated with 16 µM FRET-peptide PEK-054 (FITC-NleKKKKVLPIQLNAATDK-KDbc) [20] in the presence of varying concentrations of either protease inhibitors or lethal toxin formation inhibitors including 4-bromobenzaldehyde *N*-(2,6-dimethylphenyl) semicarbazone (EGA), calpain-inhibitor MDL281700, furin inhibitor II (hexa-d-arginine (D6R)), EDTA, ZnCl_2_, N-ethylmaleimide (NEM) and aprotinin in HEPES buffer (20 mM HEPES, 0.05% Tween-20, pH 8.2). To study the effect of metal ions on PA_83_ proteolytic activity, 20 µg/mL PA_83_ was incubated with 16 µM FRET-peptide PEK-054 in HEPES buffer (20 mM HEPES, 0.05% Tween-20, pH 8.2) supplemented with 1 mM EDTA. Next, 5 mM CaCl_2_ or 5 mM MgCl_2_ was added to the reaction to restore potential protease activity. All above mentioned chemicals were purchased from Sigma Aldrich. Protease activity was defined as a percentage of the control (PA_83_ activity without inhibitor or metal ions).

### LC MS/MS

Prior to LC MS/MS analysis 10 µg PA_83_ was digested with 0.5 µg trypsin (Difco, BD Biosciences) for 3 hr at 37°C. The trypsinized sample was analyzed by MS/MS using the data dependent LC-auto MS/MS mode. The ten most abundant ions (charge states 2+, 3+ and 4+) in the MS spectrum (300–1300 m/z, 2Hz) of eluted peptides were selected for data dependent MS/MS analysis by collision-induced dissociation using nitrogen as the collision gas. MS/MS scans were acquired over the mass range 100–2000 m/z (2–10 Hz, depending on signal intensities). Data files were exported to mzML format with MSConvert (ProteoWizard 3.0.8764), and searched with Peaks 7.5 (Bioinformatics Solutions Inc.) against a custom database consisting of NCBI RefSeq v77 protein sequences from *Homo sapiens, Bos taurus, Ovis aries* and > 400 selected human pathogenic, commensal and near-neighbor bacterial species. Precursor tolerance was 40 ppm and product ion tolerance was set to 0.04 Da. Semi-tryptic peptides were allowed with up to one missed enzymatic cleavage. Oxidation of methionine was set as variable modification. A PSM score threshold was selected corresponding to a false discovery rate of 0.1%, and protein groups required a minimum of two unique peptides. The resulting FDR at the peptide and protein level were both < 1%.

### SDS-PAGE

PA_83_ protein (2 µg) was mixed 1:1 with 2x Laemmli sample buffer (Biorad), incubated for 5 min at 95°C and loaded onto a 10% SDS-PAGE gel. After electrophoresis for 1 hr at 22 mA (max. 160 V) the gel was stained overnight with PageBlue staining solution (Fisher Scientific) and washed two times to visualize bands.

### Specificity testing PA_83_

20 µg/mL PA_83_ was incubated with 15 µg/mL *B. anthracis* protective antigen antibody-[HRP] (α-PA, Novusbio, BAP0102), 15 µg/mL human IgG antibody-[HRP] (α-human IgG, Sigma Aldrich, A0170) or PBS (solvent of the used antibodies) in HEPES buffer for 5 hr at room temperature. Next, 1 µL PEK-054 (800 µM) was added to the reactions and increase in fluorescence was measured at 37°C for 60 min as described above.

## Abbreviations

PA: Protective antigen
LF: Lethal factor
EF: Edema factor
TEM8: tumor endothelial marker 8
CMG2: capillary morphogenesis gene 2
MAPK: mitogen-activated protein kinase
vWA: von Willebrand factor type A
D6R: hexa-d-arginine
FRET: fluorescence resonance energy transfer

## References

[1] Glinert I, Bar-David E, Sittner A, et al Revisiting the concept of targeting only *Bacillus anthracis* toxins as a treatment for Anthrax. Antimicrob Agents Chemother. 2016;60:4878–4885. 27270276 10.1128/AAC.00546-16 PMC4958181

[2] McComb RC, Martchenko M. Neutralizing antibody and functional mapping of *Bacillus anthracis* protective antigen-the first step toward a rationally designed anthrax vaccine. Vaccine. 2016;34:13–19. 26611201 10.1016/j.vaccine.2015.11.025

[3] Bradley KA, Mogridge J, Mourez M, et al Identification of the cellular receptor for anthrax toxin. Nature. 2001;414:225–229. 11700562 10.1038/n35101999

[4] Scobie HM, Rainey GJ, Bradley KA, et al Human capillary morphogenesis protein 2 functions as an anthrax toxin receptor. Proc Natl Acad Sci USA. 2003;100:5170–5174. 12700348 10.1073/pnas.0431098100 PMC154317

[5] Friebe S, van der Goot FG, Burgi J The ins and outs of anthrax toxin. Toxins (Basel). 2016;8:69. 10.3390/toxins8030069 PMC4810214 26978402

[6] Bromberg-White J, Lee CS, Duesbery N Consequences and utility of the zinc-dependent metalloprotease activity of anthrax lethal toxin. Toxins (Basel). 2010;2:1038–1053. 22069624 10.3390/toxins2051038 PMC3153234

[7] Roux PP, Blenis J ERK and p38 MAPK-activated protein kinases: a family of protein kinases with diverse biological functions. Microbiol Mol Biol Rev. 2004;68:320–344. 15187187 10.1128/MMBR.68.2.320-344.2004 PMC419926

[8] Leppla SH Anthrax toxin edema factor: a bacterial adenylate cyclase that increases cyclic AMP concentrations of eukaryotic cells. Proc Natl Acad Sci USA. 1982;79:3162–3166. 6285339 10.1073/pnas.79.10.3162 PMC346374

[9] Bradley KA, Mogridge J, Jonah G, et al Binding of anthrax toxin to its receptor is similar to alpha integrin-ligand interactions. J Biol Chem. 2003;278:49342–49347. 14507921 10.1074/jbc.M307900200

[10] Rainey GJ, Wigelsworth DJ, Ryan PL, et al Receptor-specific requirements for anthrax toxin delivery into cells. Proc Natl Acad Sci USA. 2005;102:13278–13283. 16141341 10.1073/pnas.0505865102 PMC1201603

[11] Fu S, Tong X, Cai C, et al The structure of tumor endothelial marker 8 (TEM8) extracellular domain and implications for its receptor function for recognizing anthrax toxin. PLoS One. 2010;5:e11203. 20585457 10.1371/journal.pone.0011203 PMC2887854

[12] Petosa C, Collier RJ, Klimpel KR, et al Crystal structure of the anthrax toxin protective antigen. Nature. 1997;385:833–838. 9039918 10.1038/385833a0

[13] Ramey JD, Villareal VA, Ng C, et al Anthrax toxin receptor 1/tumor endothelial marker 8: mutation of conserved inserted domain residues overrides cytosolic control of protective antigen binding. Biochemistry. 2010;49:7403–7410. 20690680 10.1021/bi100887w PMC2942075

[14] Podsiadlo P, Komiyama T, Fuller RS, et al Furin inhibition by compounds of copper and zinc. J Biol Chem. 2004;279:36219–36227. 15140896 10.1074/jbc.M400338200

[15] Remacle AG, Gawlik K, Golubkov VS, et al Selective and potent furin inhibitors protect cells from anthrax without significant toxicity. Int J Biochem Cell Biol. 2010;42:987–995. 20197107 10.1016/j.biocel.2010.02.013 PMC2862824

[16] Sarac MS, Peinado JR, Leppla SH, et al Protection against anthrax toxemia by hexa-d-arginine *in vitro* and *in vivo*. Infect Immun. 2004;72:602–605. 14688144 10.1128/IAI.72.1.602-605.2004 PMC343991

[17] Opal SM, Artenstein AW, Cristofaro PA, et al Inter-alpha-inhibitor proteins are endogenous furin inhibitors and provide protection against experimental anthrax intoxication. Infect Immun. 2005;73:5101–5105. 16041026 10.1128/IAI.73.8.5101-5105.2005 PMC1201260

[18] Gillespie EJ, Ho CL, Balaji K, et al Selective inhibitor of endosomal trafficking pathways exploited by multiple toxins and viruses. Proc Natl Acad Sci USA. 2013;110:E4904–12. 24191014 10.1073/pnas.1302334110 PMC3864319

[19] Jeong SY, Martchenko M, Cohen SN Calpain-dependent cytoskeletal rearrangement exploited for anthrax toxin endocytosis. Proc Natl Acad Sci USA. 2013;110:E4007–15. 24085852 10.1073/pnas.1316852110 PMC3801034

[20] Cummings RT, Salowe SP, Cunningham BR, et al A peptide-based fluorescence resonance energy transfer assay for *Bacillus anthracis* lethal factor protease. Proc Natl Acad Sci USA. 2002;99:6603–6606. 11997440 10.1073/pnas.062171599 PMC124449

[21] Molhoek EM, den Hertog AL, de Vries AM, et al Structure-function relationship of the human antimicrobial peptide LL-37 and LL-37 fragments in the modulation of TLR responses. Biol Chem. 2009;390:295–303. 19166322 10.1515/BC.2009.037

[22] Kaman WE, Hulst AG, van Alphen PT, et al Peptide-based fluorescence resonance energy transfer protease substrates for the detection and diagnosis of *Bacillus* species. Anal Chem. 2011;83:2511–2517. 21370823 10.1021/ac102764v

[23] Kaman WE, Voskamp-Visser I, de Jongh DM, et al Evaluation of a d-amino-acid-containing fluorescence resonance energy transfer peptide library for profiling prokaryotic proteases. Anal Biochem. 2013;441:38–43. 23850560 10.1016/j.ab.2013.06.015

